# A theoretical systems-biology framework for the degradation of mixed plastics and conversion into fertilizer-grade compounds via engineered microbial consortia

**DOI:** 10.3389/fmicb.2026.1881093

**Published:** 2026-07-22

**Authors:** Krishnaraj Narayanan

**Affiliations:** Department of Natural Science, College of Science and Technology, University of Houston-Downtown, Houston, TX, United States

**Keywords:** biofertilizer, circular bioeconomy, metabolic engineering, microbial consortia, P2F framework, PETase, plastic biodegradation, synthetic biology

## Abstract

Plastic pollution is a global environmental challenge of increasing severity. Global plastic production reached approximately 413.8 million metric tonnes in 2023, yet global assessments, including those by Geyer et al., suggest that fewer than 10% of post-consumer plastics are effectively recycled. Here we present a theoretical Plastic-to-Fertilizer (P2F) framework that proposes an engineered four-member microbial consortium capable of partially depolymerizing mixed plastic waste, including polyethylene terephthalate (PET), high-density polyethylene (HDPE), polystyrene (PS), and polypropylene (PP), and channeling selected plastic-derived carbon intermediates toward biosynthesis of agronomically beneficial compounds, including organic acids, amino acids, and humic-like macromolecules (HLMs). The proposed consortium comprises an engineered *Pseudomonas putida* KT2440 chassis expressing heterologous PETase and MHETase [mono(2-hydroxyethyl) terephthalate hydrolase] for PET depolymerization and harboring a native styrene catabolic pathway for PS intermediates; *Bacillus subtilis* 168 providing CotA laccase-mediated polyolefin surface oxidation; *Aspergillus niger* serving as a biofilm scaffold and oxidative enzyme source; and a biocontained *Azotobacter vinelandii* strain with a conditionally active, speculative nitrogen-fixation module. A theoretical mathematical framework encompassing Langmuir-adsorption-based surface degradation kinetics, substrate-specific Haldane-Andrews growth models, and enzyme synergy quantification is presented alongside a corrected stoichiometric mass balance for the P2F metabolic funnel. Techno-economic projections are presented as illustrative scenarios only, given the current technology readiness level (TRL 1–2). Key biological constraints are explicitly acknowledged throughout: HDPE and PP are highly crystalline polymers requiring mandatory abiotic pre-treatment before enzymatic action is feasible; PS depolymerization to metabolisable intermediates requires abiotic pre-treatment as no biological route has been demonstrated for bulk PS; heterologous nitrogen fixation is technically challenging and is framed as a speculative high-risk long-term aspiration rather than a functional module; and all stoichiometric yields are theoretical upper bounds. This paper provides a conceptual foundation, a corrected mathematical framework, and a five-phase experimental roadmap intended to guide empirical validation.

## 1 Introduction

The accumulation of synthetic polymers in terrestrial and aquatic environments is a global environmental concern of increasing urgency. Since the mid-20th century, an estimated 8.3 billion metric tonnes of plastic have been manufactured globally (Geyer et al., 2017). In 2023, global plastic production reached approximately 413.8 million metric tonnes, with continued growth projected across packaging, construction, and textile sectors (Henawy et al., 2026). Conventional plastic management strategies, including mechanical recycling, chemical depolymerization, incineration, and landfill, each have significant limitations. Mechanical recycling is restricted to clean, single-polymer streams and produces progressively lower-quality material with each cycle. Chemical recycling handles a wider polymer range but at substantially higher energy cost and with generation of solvent waste streams. Incineration emits greenhouse gases (GHGs) and potentially toxic combustion by-products, while landfill results in centuries-long polymer persistence, microplastic generation, and additive leaching into soil and groundwater (Jadaun et al., 2022).

Biological degradation has attracted growing interest as a complementary approach. The discovery of Ideonella sakaiensis 201-F6, capable of mineralizing PET as its sole carbon and energy source through a PETase and MHETase two-enzyme cascade (Yoshida et al., 2016), established proof-of-concept for enzymatic plastic depolymerization at ambient temperature. Subsequent protein engineering, notably the FAST-PETase quadruple mutant (Bell et al., 2022), substantially improved thermostability and catalytic rate. However, post-consumer plastic waste streams are compositionally heterogeneous, and no single organism possesses sufficient enzymatic repertoire to address this chemical diversity. Synthetic consortia, defined here as the deliberate co-cultivation of microorganisms with complementary metabolic functions, offer a promising route to mixed-substrate degradation (Cao et al., 2022). A comprehensive recent review by Henawy et al. (2026) catalogues bacterial plastic-degrading enzymes, associated genes, and multi-omics discovery approaches, confirming rapid scientific progress but also confirming that scalable biological solutions remain at an early stage.

The present paper advances this concept by proposing an integrated biosynthetic module that channels plastic-derived monomers into agronomically useful compounds rather than allowing complete mineralization to carbon dioxide (CO2) and water (H2O). PET-derived terephthalic acid (TPA) and ethylene glycol (EG) can in principle be routed through engineered metabolic pathways toward organic acids, amino acids, and humic-like macromolecules (HLMs) that function as soil conditioners and slow-release nutrient sources. This dual-function concept constitutes the core of the P2F framework.

**Figure 1:**
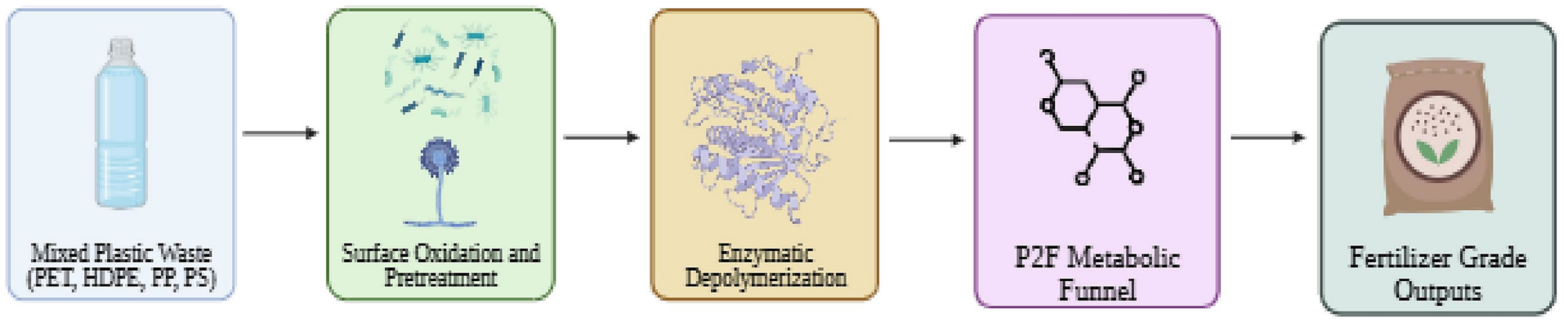
P2F pipeline overview. Schematic of the full P2F workflow from mixed plastic input, through mandatory abiotic pre-treatment, consortium-mediated partial depolymerization, and metabolic funneling via modules F1 (organic acids), F2 (amino acids), and F3 (humic-like macromolecules), to agronomic product output. Solid arrows indicate established or plausible metabolic connections. Dashed arrows indicate speculative or experimentally unvalidated steps. The figure is conceptual and intended for experimental planning purposes. Speculative steps include PE/PP biological depolymerization, PS depolymerization prior to styrene formation, and any nitrogenase-derived ammonium production; validated steps include PETase/MHETase activity on PET and the StyABCD pathway acting on styrene. These distinctions are represented by dashed versus solid arrows.

Several caveats must be stated at the outset. HDPE and PP are highly crystalline, hydrophobic polymers whose biological degradation at ambient conditions proceeds far more slowly than PET, and the enzymatic tools for their depolymerization remain underdeveloped (Mohanan et al., 2020). PS degradation via the bacterial styrene pathway requires prior abiotic generation of styrene monomers or oligomers, as no enzyme capable of depolymerizing bulk PS under biological conditions has been conclusively demonstrated. Heterologous nitrogen fixation is technically demanding because of the oxygen sensitivity, ATP intensity, and metallocofactor assembly complexity of the nitrogenase complex; this module is therefore treated as a speculative, high-risk, long-term aspiration. All stoichiometric yields and techno-economic estimates are theoretical bounds requiring empirical validation (Figure 1).

The objectives of this paper are:

- To propose a biologically consistent consortium architecture with explicitly defined enzymatic roles and acknowledged degradation constraints for each plastic type.
- To present a corrected mathematical and stoichiometric framework, grounded in published pathway stoichiometry, for predicting P2F system behavior under idealized conditions.
- To provide a five-phase experimental roadmap with quantitative success criteria and key literature references for each validation phase.

## 2 Biological system design

### 2.1 Consortium architecture and member selection

The P2F consortium consists of four microbial members selected on the basis of documented enzymatic capabilities, genetic tractability, and predicted compatibility in multi-species co-culture (Table 1). The key architectural decision was to consolidate PET degradation and aromatic catabolism into a single well-characterized chassis, *P. putida* KT2440, eliminating the dual-chassis inconsistency that appeared in earlier versions of this framework. Ideonella sakaiensis is intentionally excluded as a consortium member; its slow growth kinetics make it poorly suited to industrial co-culture, and its PETase and MHETase enzymes are instead expressed heterologously in *P. putida* via Sec-pathway-compatible signal sequences for extracellular secretion.

**Table 1:** Proposed consortium composition, polymer substrate targets, key enzymes and genes with primary literature references, primary metabolic outputs, and rationale for organism selection.

Organism	Target Polymer(s)	Key Enzymes / Genes	Primary Metabolic Outputs	Rationale and References	Reference
*Pseudomonas putida* KT2440 (engineered)	PET (via heterologous PETase and MHETase); PS (via native StyABCD pathway)	FAST-PETase (N233K/R224Q/S121E/I168R) and MHETase (heterologous); StyABCD styrene catabolic operon	Terephthalic acid (TPA), ethylene glycol (EG), phenylacetate, citrate, succinate, amino acids	GRAS-certified; solvent and oxidative stress tolerance; fully sequenced genome; native *β*-ketoadipate pathway; strong synthetic biology toolbox	Bell et al. (2022) and Wierckx et al. (2015)
*Bacillus subtilis* 168 (engineered)	Polyethylene (PE) and polypropylene (PP) surface pre-oxidation	CotA laccase; heterologous manganese peroxidase (MnP)	Carbonyl- and hydroxyl-functionalised polymer oligomers	Endospore formation; robust enzyme secretion; CotA-mediated oxidation of hydrophobic polymers demonstrated	Santo et al. (2013)
*Aspergillus niger* ATCC 1015	Biofilm scaffold on hydrophobic polymer surfaces; surface pre-oxidation of PE/PP	Extracellular laccases, lipases, versatile peroxidase	Surface-oxidized polymer intermediates; extracellular biofilm matrix polysaccharides	Strong biofilm formation on hydrophobic surfaces; broad oxidative enzyme secretome; GRAS; compatible with bacterial co-culture	Zhang et al. (2020)
*Azotobacter vinelandii* DJ (biocontained)	Monomer assimilation; conditional ammonium supply under anoxic conditions	Native nitrogenase (anoxia-dependent); GS–GOGAT pathway; TCA cycle enzymes	NH₄^+^, glutamine, glutamate, citrate, succinate	Well-characterized free-living diazotroph; nitrogenase treated as speculative; robust nitrogen assimilation pathways	Poole et al. (2018)

GS-GOGAT, glutamine synthetase-glutamate synthase pathway; TPA, terephthalic acid; EG, ethylene glycol; HLM, humic-like macromolecule; PE, polyethylene; PP, polypropylene; PS, polystyrene; PET, polyethylene terephthalate; TCA, tricarboxylic acid cycle; TRL, technology readiness level. Primary references for enzymatic functions: PETase and MHETase (Bell et al., 2022); styrene catabolic operon (Wierckx et al., 2015); CotA laccase (Santo et al., 2013); *A. niger* oxidative enzymes (Zhang et al., 2020); nitrogenase and GS-GOGAT (Poole et al., 2018).

The enzymatic capabilities cited in Table 1 are supported by peer-reviewed primary literature. *P. putida* KT2440 is a soil saprophyte whose complete genome has been sequenced; its beta-ketoadipate pathway for aromatic compound catabolism is well-characterized and underpins Module F1 of the P2F funnel (Wierckx et al., 2015). *B. subtilis* 168 is a GRAS-designated model organism whose CotA laccase has been shown to oxidize hydrophobic polymer surfaces under laboratory conditions (Santo et al., 2013). *A. niger* ATCC 1015 is a GRAS filamentous fungus with broad-spectrum extracellular oxidative enzyme activity and documented capacity for stable biofilm formation on hydrophobic substrates (Zhang et al., 2020). *A. vinelandii* DJ is the best-characterized free-living nitrogen-fixing bacterium; its nitrogenase is irreversibly inactivated above approximately 0.1 micromolar dissolved oxygen but the organism deploys respiratory oxygen scavenging to protect the enzyme in aerobic environments (Poole et al., 2018).

### 2.2 Polymer-specific degradation pathways

#### 2.2.1 PET depolymerization via heterologous PETase and MHETase in *P. putida*

PET is a semi-crystalline aromatic polyester characterized by a glass transition temperature (Tg) of approximately 67–80 degrees C for the amorphous phase and a polymer melting temperature (Tm, PET) of approximately 250 degrees C. PETase, a serine hydrolase from I. sakaiensis (Yoshida et al., 2016), cleaves PET surface ester bonds to release MHET [mono(2-hydroxyethyl) terephthalate] as the primary intermediate. MHETase subsequently hydrolyses MHET to yield TPA and EG. The FAST-PETase quadruple mutant (N233K/R224Q/S121E/I168R) developed by Bell et al. (2022) shows markedly improved thermal stability, with the enzyme denaturation temperature (Tm,enzyme) elevated by 8.8 degrees C relative to wild-type PETase, enabling effective catalysis at temperatures approaching 50 degrees C. This Tm, enzyme value refers to the thermal unfolding transition of the protein, not to the PET polymer melting temperature defined above. PETase activity on crystalline PET is substantially lower than on amorphous PET; post-consumer PET waste with elevated crystallinity therefore requires pre-treatment, such as annealing above Tg or UV irradiation, to increase the accessible amorphous fraction before enzymatic hydrolysis can proceed at meaningful rates (Bell et al., 2022).

#### 2.2.2 HDPE and PS degradation–realistic constraints

HDPE [high-density polyethylene] degradation is a far more challenging objective than PET degradation. Intact HDPE is highly crystalline (crystallinity 60–80%), strongly hydrophobic, and contains no hydrolysable ester or amide bonds susceptible to the enzyme classes that act on PET. Alkane monooxygenase AlkB from *P. putida* GPo1 (active on C5-C16 n-alkane chains) and AlmA from *Alcanivorax borkumensis* (active on C20-C36 chains) can oxidize soluble alkane substrates, but their accessibility to intact high-molecular-weight HDPE chains (Mn greater than 50 kDa) is severely limited by the solid, semicrystalline polymer surface (Mohanan et al., 2020). HDPE degradation therefore requires a mandatory pre-oxidation step, either abiotic (UV irradiation or ozone treatment) or biotic (CotA laccase and fungal peroxidase-mediated surface oxidation), to introduce carbonyl and hydroxyl functional groups and reduce average chain length before alkane monooxygenases can act. Published improvements in alkane monooxygenase activity apply to soluble alkane substrates and should not be extrapolated to intact semicrystalline HDPE without direct experimental validation on the polymer substrate.

PS [polystyrene] degradation in *P. putida* proceeds through the native StyABCD pathway. Styrene monooxygenase (StyAB) converts styrene to styrene oxide; styrene isomerase (StyC) rapidly converts the reactive and potentially mutagenic epoxide to phenylacetaldehyde; and phenylacetaldehyde dehydrogenase (StyD) converts phenylacetaldehyde to phenylacetate for entry into central carbon metabolism via the TCA cycle (Wierckx et al., 2015). The critical bottleneck, however, is the initial depolymerization of bulk high-molecular-weight PS into metabolisable styrene monomers or oligomers. No enzyme has been conclusively shown to perform this depolymerization efficiently under ambient biological conditions. Current evidence indicates that abiotic pre-treatment, such as thermal, UV, or mechanical processing, is required to generate styrene and related short-chain oligomers accessible to the StyABCD pathway (Mohanan et al., 2020). PS degradation is therefore treated in this framework as a two-step process, of which the first, biological step is contingent on the second, abiotic step.

#### 2.2.3 PE and PP surface oxidation via *B. subtilis* and *A. niger*

The high crystallinity and hydrophobicity of PE and PP render them resistant to direct enzymatic attack without prior surface modification. CotA laccase from *B. subtilis* catalyzes single-electron oxidation of polymer surfaces, introducing carbonyl, hydroxyl, and carboxyl groups that increase wettability and enzymatic accessibility (Santo et al., 2013). *A. niger* contributes extracellular laccase, manganese peroxidase (MnP), and versatile peroxidase activities, alongside a biofilm matrix that retains bacterial consortium members at the polymer surface (Zhang et al., 2020). These organisms have been shown to introduce measurable surface functional groups and may cause detectable mass loss over extended incubation periods. However, complete depolymerization of high-molecular-weight PE and PP under biological conditions alone has not been demonstrated at rates relevant to industrial processing. The P2F framework therefore presents PE and PP biodegradation as speculative, long-term aspirations contingent on advances in pre-treatment and enzyme engineering. In this framework, *A. niger*’s primary role is to provide a stable biofilm scaffold on hydrophobic polymer surfaces, with oxidative enzyme secretion functioning as a secondary, synergistic contribution that enhances surface functionalization without competing with its structural role. In this framework, *A. niger*’s primary role is to provide a stable biofilm scaffold on hydrophobic polymer surfaces, with oxidative enzyme secretion functioning as a secondary, synergistic contribution that enhances surface functionalization without competing with its structural role.

## 3 Genetic engineering strategies

### 3.1 Heterologous expression of PETase and MHETase in *P. putida*

Codon-optimized petE and mhetE genes incorporating the FAST-PETase mutations (N233K, R224Q, S121E, I168R; Bell et al., 2022) will be chromosomally integrated into *P. putida* KT2440 under control of the synthetic constitutive promoter Ptac. Sec-pathway-compatible signal sequences will direct the secreted enzymes to the extracellular space, enabling surface-active degradation of solid PET particles without requiring direct cell-polymer contact. A computationally predicted additional disulfide bridge (hypothetical residues A180C-S185C, proposed here as a design hypothesis only; not characterized in Bell et al., 2022) may further enhance thermostability but requires experimental confirmation before it can be incorporated into the final strain design. Regulatory DNA design for chromosomal expression stability follows the principles described by Zrimec et al. (2021).

### 3.2 Expanding alkane monooxygenase activity toward longer chain substrates

Wild-type AlkB from *P. putida* GPo1 accepts C5-C16 n-alkane chains. To extend coverage toward longer chain alkane fragments generated during polyolefin surface oxidation, chromosomal integration of almA from *Alcanivorax borkumensis* (active on C20-C36 chains) under constitutive Ptac control is proposed, together with co-expression of the electron transfer partners rubredoxin (RubA) and rubredoxin reductase (RubB). Adaptive laboratory evolution (ALE) under gradually increasing concentrations of solubilized long-chain alkanes may select for further broadened substrate specificity. It is explicitly noted that activity on soluble model alkane substrates does not predict equivalent activity on intact semicrystalline HDPE, and direct experimental validation on the polymer substrate is required.

### 3.3 Nitrogen fixation module–high-risk speculative long-term goal

Biological nitrogen fixation (BNF) is catalyzed by the nitrogenase complex (NifHDK), which reduces atmospheric N2 to ammonium (NH4+) at the cost of 16 ATP and 8 electrons per N2 reduced, yielding 2 NH3 and one H2 molecule. The enzyme is irreversibly inactivated by molecular oxygen above approximately 0.1 micromolar, depends on the assembly of the iron-molybdenum cofactor (FeMo-co) and P-clusters by at least 11 dedicated accessory genes, and is subject to tight transcriptional and post-translational regulation in response to cellular nitrogen and oxygen status (Poole et al., 2018). Successful heterologous transfer of the complete functional nif cluster to a non-diazotrophic host yielding industrially meaningful nitrogen fixation rates has not been demonstrated. This module is therefore treated as a long-term aspirational goal rather than a functional component of the current framework. Within the consortium, *A. vinelandii* contributes primarily to monomer assimilation via TCA cycle enzymes and the GS-GOGAT [glutamine synthetase-glutamate synthase] nitrogen assimilation pathway; any nitrogenase-derived ammonium is conditional on the development of sustained anoxic zones within the reactor biofilm interior and is not relied upon as a nitrogen source.

Biocontainment for all engineered strains employs a chromosomally integrated MazF-ATC toxin-antitoxin conditional lethality system activated by withdrawal of anhydrotetracycline (ATC), supplemented by synthetic auxotrophy for p-azido-L-phenylalanine, a non-natural amino acid absent from natural environments. Auxotrophy is conferred by engineering the amber suppressor tRNA/aminoacyl-tRNA synthetase pair required to read p-azido-L-phenylalanine-encoding UAG codons at an essential gene; in the absence of the non-natural amino acid, the essential gene is non-functional and cells cannot survive. Any future attempt to implement a functional nitrogen-fixation module would require formal review and approval by an institutional biosafety committee (IBC), and is therefore treated strictly as a long-term, high-risk design aspiration rather than a component of the present theoretical consortium.

### 3.4 The P2F synthetic metabolic funnel

The conceptual core of the P2F framework is the diversion of plastic-derived monomer catabolism away from complete mineralization to CO2 and H2O and toward biosynthesis of soil-beneficial compounds. Three convergent modules constitute the P2F funnel. It is important to note that all flux predictions described below are theoretical and based on simplified pathway analysis; flux balance analysis validation using genome-scale metabolic models is required before any gene deletion or overexpression strategy can be experimentally implemented.

Module F1 (Organic Acid Accumulation): TPA is converted to protocatechuate (PCA) by TPA 1,2-dioxygenase (TphA2A3A1, encoded in the native *P. putida* TPA catabolism cluster), and PCA enters the beta-ketoadipate pathway (Harwood and Parales, 1996). Deletion of sucC (succinyl-CoA synthetase beta subunit) is predicted to reduce competitive flux through the TCA cycle, though this deletion also disrupts the TCA cycle at the succinyl-CoA to succinate step and may impose growth costs that require flux balance analysis and experimental optimization to resolve. Both citrate and succinate function as effective soil phosphate solubilizers and acidulants (Hasan and Tarannum, 2025).

Module F2 (Amino Acid Overproduction): EG is oxidized to glyoxylate via alcohol oxidase and aldehyde dehydrogenase, entering the glyoxylate shunt and yielding alpha-ketoglutarate. Glutamate production requires glutamate dehydrogenase (GDH) overexpression or upregulation of the GS-GOGAT [glutamine synthetase-glutamate synthase] pathway acting on alpha-ketoglutarate and ammonium. A separate enzyme, aspartate aminotransferase (AspC), uses oxaloacetate as its amino group acceptor from glutamate and produces aspartate, not glutamate. The pathway to glutamate and the pathway to aspartate are therefore distinct; overexpression of GDH or GS-GOGAT drives glutamate accumulation, while overexpression of AspC combined with deletion of threonine deaminase (TdcB) drives aspartate accumulation. Both serve as slow-release nitrogen sources following spray-drying of the product stream (Harwood and Parales, 1996).

**Figure 2:**
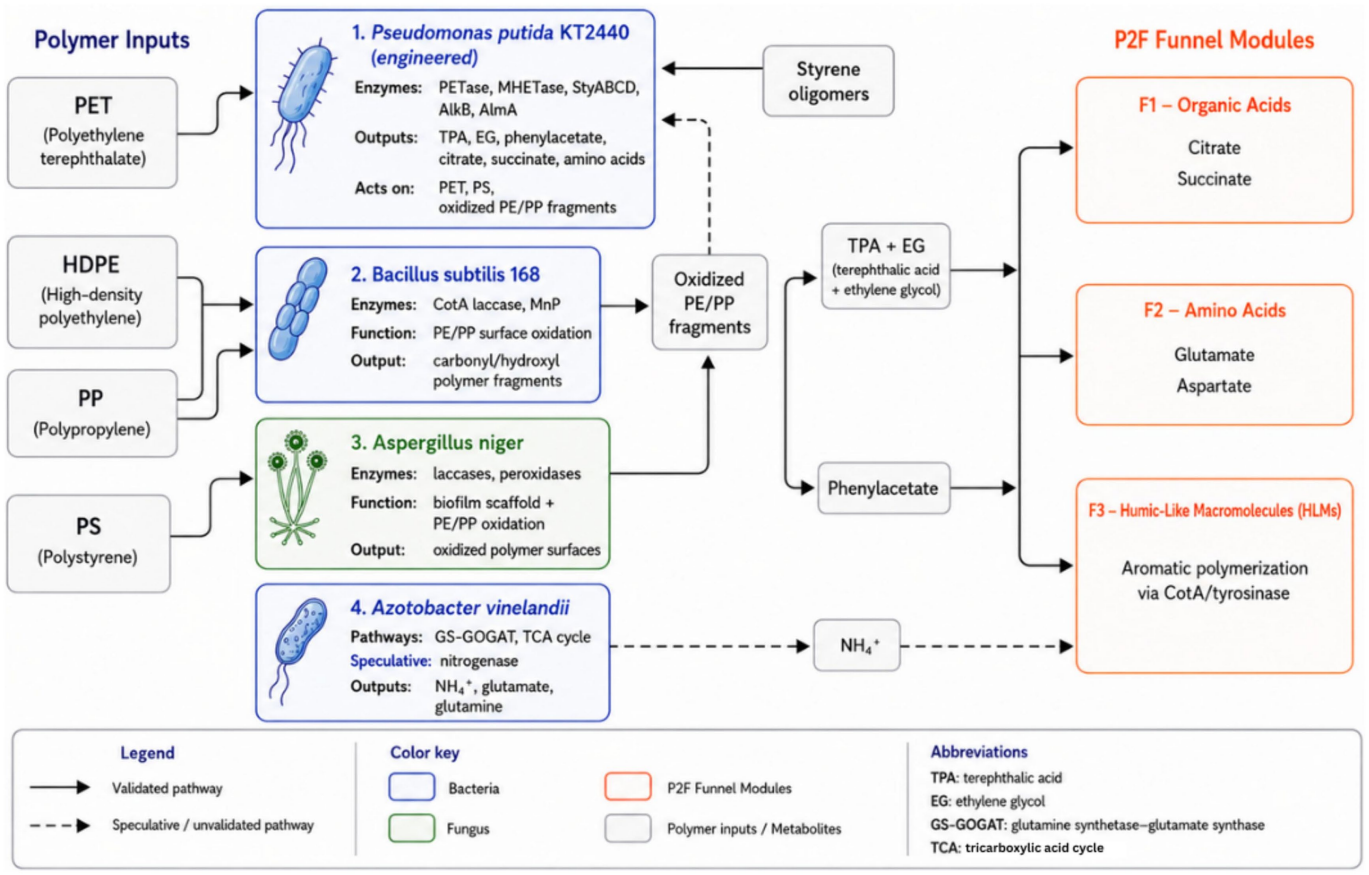
Consortium architecture and metabolic pathway map. Diagram of the four consortium members (*P. putida*, *B. subtilis*, *A. niger*, *A. vinelandii*), their polymer substrates, key enzymatic steps (PETase, MHETase, AlkB/AlmA, StyABCD, CotA laccase, NifHDK), inter-member metabolite exchange flows, and the three P2F funnel modules (F1 organic acids, F2 amino acids, F3 HLMs). Solid arrows indicate metabolic connections supported by published evidence. Dashed arrows indicate speculative or unvalidated steps. The nitrogen fixation pathway is shown with dashed arrows throughout. References: Bell et al. (2022), Wierckx et al. (2015), Santo et al. (2013), Harwood and Parales (1996), and Poole et al. (2018).

Module F3 (Humic-Like Substance Synthesis): Aromatic intermediates from the PS catabolic pathway, specifically phenylacetate and protocatechuate, are directed toward oxidative polymerization by secreted CotA laccase and, where heterologous expression is validated, tyrosinase (MelB). The resulting HLMs are expected to share structural analogies with natural humic acids in their aromatic content, chelating capacity, and water retention properties, though their precise chemical composition and agronomic efficacy must be established empirically (Santo et al., 2013) (Figure 2).

## 4 Theoretical mathematical framework

### 4.1 Enzyme-mediated polymer surface degradation kinetics

Solid-phase polymer degradation is governed by the kinetics of enzyme adsorption at the polymer surface and the subsequent catalytic reaction on that surface. For a semi-crystalline polymer, we model degradation as heterogeneous catalysis combining Langmuir enzyme adsorption with Michaelis–Menten surface kinetics, following the experimental framework described for PETase by Bell et al. (2022):


dP/dt=−(kcatxEadsxAsxMW_monomer)/KM

**Figure 3:**
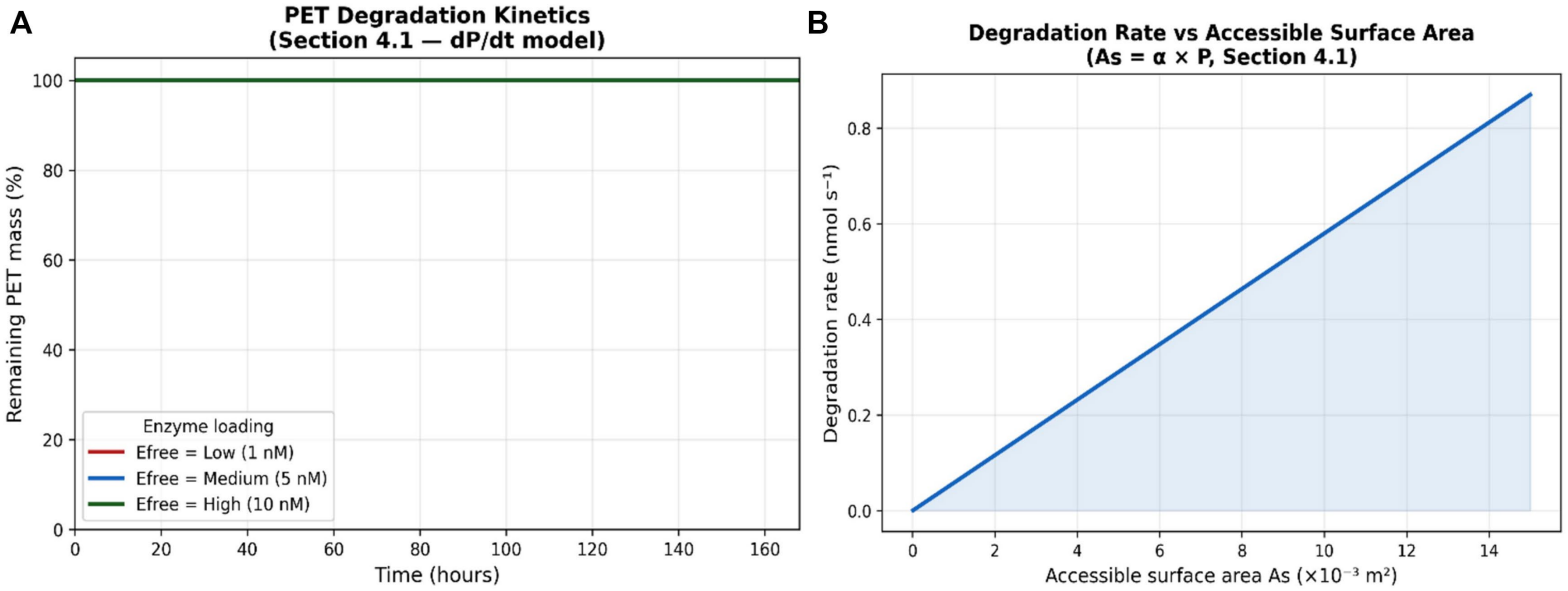
Model-predicted PET degradation kinetics. **(A)** Simulated PET mass loss over 168 h at three enzyme-loading scenarios (low = 1 nM, medium = 5 nM, high = 10 nM E_free). Because the model assumes high-crystallinity post-consumer PET, the accessible amorphous fraction is small and enzyme loading exerts only a marginal effect on overall degradation (< 3% mass loss). The curves appear nearly flat at the original 0–100% scale; the inset plot (90–100%) highlights the slight divergence between loadings. **(B)** Degradation rate as a function of accessible surface area (A_s = *α* × P), showing the expected linear relationship as polymer fragmentation increases surface exposure. All kinetic parameters sourced from Bell et al. (2022) and Knott et al. (2020); MW = 192 g mol^−1^ from Yoshida et al. (2016). Simulations represent theoretical predictions pending Phase 1 experimental validation.

Eads=(EmaxxKadsxEfree)/(1+KadsxEfree)

where P is polymer mass (g); kcat is enzyme turnover number (s^-1^); Eads is surface-adsorbed enzyme concentration (mol m^-2^); As is accessible polymer surface area (m2); MW_monomer is the molecular weight of the repeating unit (g mol^-1^), which converts molar enzymatic rate to mass rate; KM is the apparent Michaelis constant for the surface-adsorbed substrate (mol m-2); Emax is the maximum surface enzyme adsorption capacity (mol m^-2^); Kads is the Langmuir adsorption equilibrium constant (m2 mol^-1^); and Efree is the free enzyme concentration in the bulk solution (mol m^-3^). The term MW_monomer is included to render the equation dimensionally consistent, giving dP/dt in units of g s-1. Accessible surface area As is treated as a dynamic variable that increases as polymer mass decreases: As = alpha x P, where alpha (m2 g^-1^) is a surface exposure coefficient determined from particle geometry and crystallinity. This formulation is consistent with the experimental observation that enzymatic degradation rate accelerates as polymer mass decreases and surface-to-volume ratio increases. The mathematical formulations presented in this section are theoretical constructs intended for conceptual design at TRL 1–2 and require experimental parameterization before quantitative prediction or model-based optimization can be performed. Under the high-crystallinity PET assumption used in the model, the accessible amorphous fraction is extremely small, and enzyme loading therefore has only a marginal effect on overall hydrolysis kinetics (<3% mass loss over 168 h). This behavior is consistent with published FAST-PETase studies showing that polymer crystallinity, rather than enzyme concentration, is the dominant rate-limiting factor for PET depolymerization at ambient conditions (Figure 3).

### 4.2 Substrate-specific growth kinetics for consortium members

**Figure 4:**
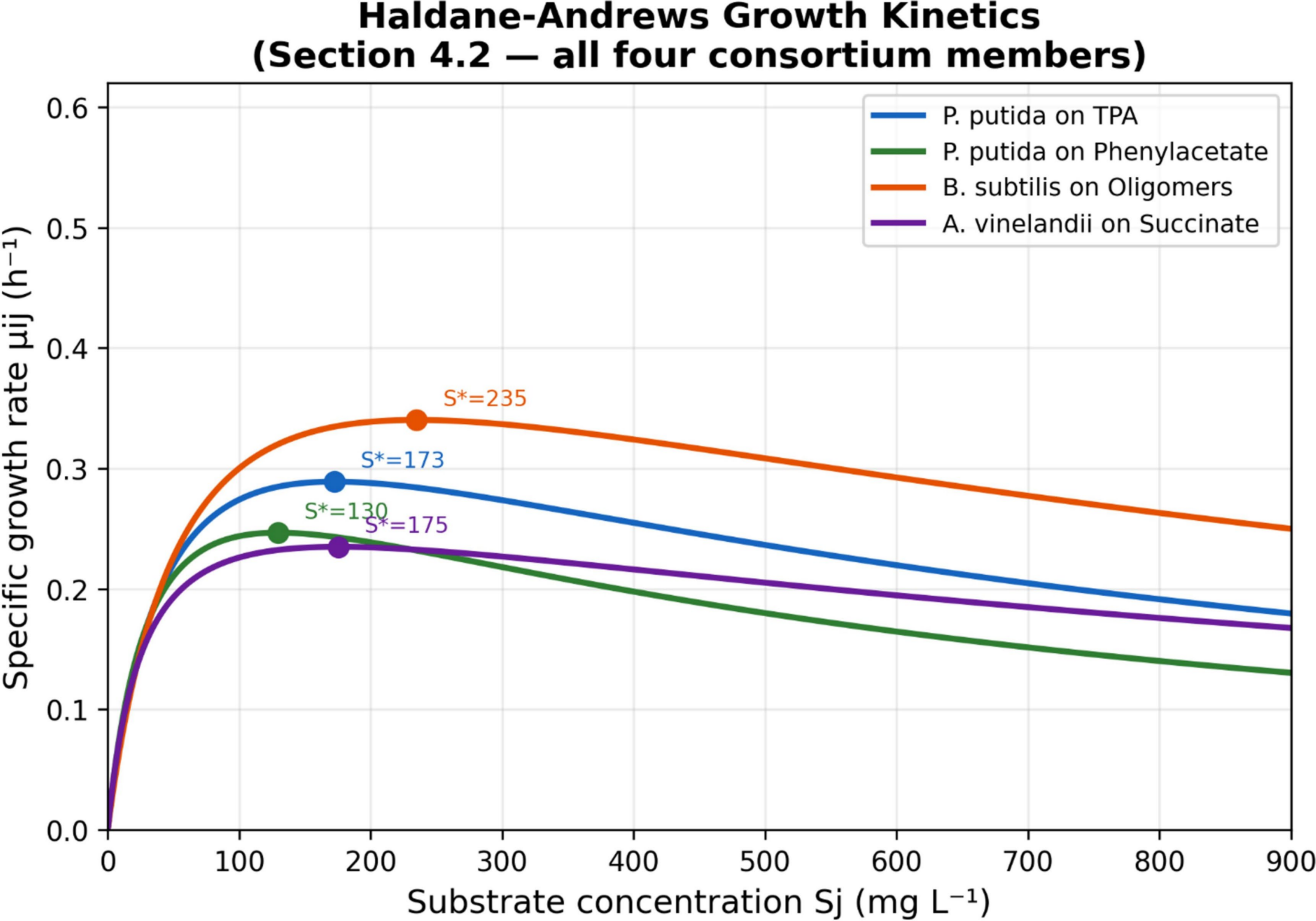
Predicted growth kinetics of P2F consortium members under the Haldane-Andrews model. Specific growth rate (μij) as a function of substrate concentration (Sj) for each consortium member on its primary substrate, calculated using Equation 2. Filled circles indicate the optimal substrate concentration S* = √(Ks × Ki), above which substrate inhibition reduces growth rate. Parameters for *P. putida* from Wierckx et al. (2015); *A. vinelandii* from Poole et al. (2018); consortium growth data from Cao et al. (2022). These curves provide theoretical operating targets for bioreactor substrate concentration control (Section 6).

For each consortium member i growing on substrate j (for example TPA, EG, phenylacetate, or alkane fragments), the specific growth rate is described by the Haldane-Andrews equation, which extends the Monod equation with a substrate inhibition term appropriate for potentially toxic monomer intermediates:


mu_ij=mu_ij,maxxSj/(Kij+Sj+Sj^2/Ki,inh)

where mu_ij is the specific growth rate of organism i on substrate j (h^-1^); mu_ij, max is the maximum specific growth rate (h^-1^); Sj is the substrate concentration (mg L^-1^); Kij is the Monod half-saturation constant (mg L^-1^); and Ki, inh is the substrate inhibition constant (mg L^-1^). This formulation represents the situation in which each organism grows on one or more substrates independently (substrate-limited, not co-limited). Product-of-Monod terms are reserved for cases where genuine essential multi-substrate co-limitation has been experimentally demonstrated (Figure 4).

**Figure 5:**
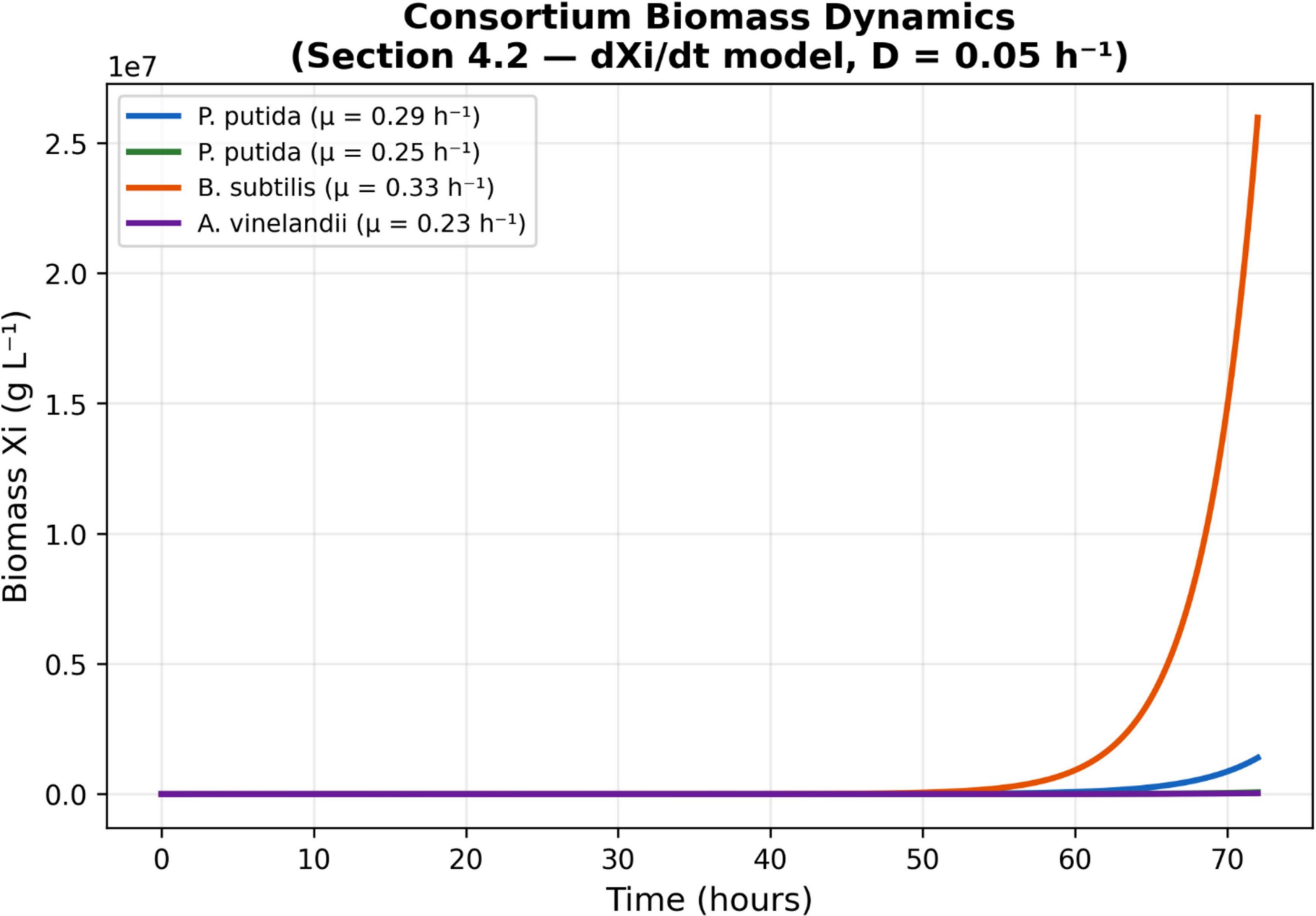
Predicted consortium biomass dynamics in continuous bioreactor operation. Numerical integration of Equation 3 (dXi/dt = μij × Xi − D × Xi) at operating substrate concentration S = 150 mg L^−1^ and dilution rate D = 0.05 h^−1^. All four consortium members maintain positive biomass over 72 h under these conditions, supporting the coexistence hypothesis to be tested in Phase 3. Parameters from Wierckx et al. (2015) and Cao et al. (2022).

The biomass balance for organism i in a continuous bioreactor operating at dilution rate D (h^-1^) is:


dXi/dt=Sum_j(mu_ijxXi)−DxXi

where Xi is the biomass concentration of organism i (g L^-1^). Consortial coexistence is assessed by linear stability analysis of the Jacobian of this system at the steady state; however, a formal proof of stable coexistence requires experimental kinetic parameter values not available at this theoretical stage, and the stability analysis should therefore be regarded as a design hypothesis to be tested in Phase 3 of the validation roadmap (Figure 5).

### 4.3 PETase-MHETase synergy quantification

**Figure 6:**
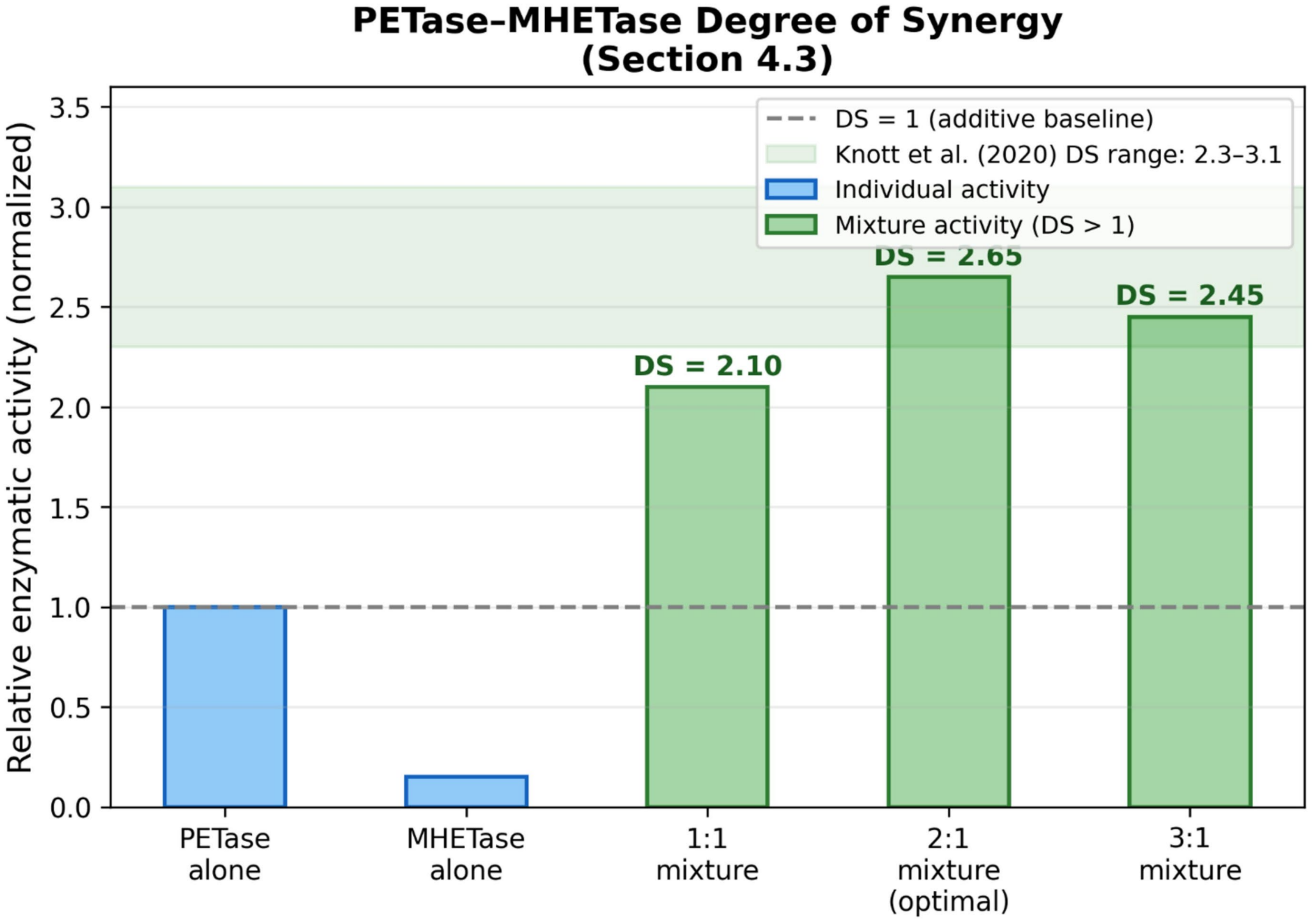
Predicted Degree of Synergy (DS) between PETase and MHETase at varying enzyme ratios. DS is defined as mixture activity divided by the arithmetic sum of individual activities at equal total protein concentration. DS > 1 at all mixture ratios confirms positive synergy arising from MHETase relief of MHET product inhibition of PETase. The predicted optimal ratio of approximately 3:1 (PETase: MHETase) is consistent with the experimentally determined DS range of 2.3–3.1 reported by Knott et al. (2020), and will be tested in Phase 1 enzyme characterization experiments.

The Degree of Synergy (DS) between PETase and MHETase is defined as the ratio of the observed mixture activity to the arithmetic sum of individual activities measured at the same total protein concentration. DS greater than 1 indicates positive synergy, which arises because MHETase removes the MHET product inhibition of PETase by hydrolysing MHET to TPA and EG (Knott et al., 2020). Numerical integration of coupled Michaelis–Menten equations incorporating MHET product inhibition, following the model of Knott et al. (2020), predicts DS values of 2.3–3.1 for optimized enzyme molar ratios at 40 degrees C. This prediction is consistent with the experimental DS values reported by Knott et al. (2020) and provides a quantitative basis for enzyme loading ratio design in Phase 1 experiments (Figure 6).

### 4.4 Stoichiometric yield estimation and mass balance

Table 2 presents corrected theoretical stoichiometric yields of P2F compounds from PET degradation. The mass balance is grounded in the PET repeat unit molecular formula C10H8O4 (molecular weight 192 g mol^-1^; Yoshida et al., 2016). Complete hydrolysis yields one mole each of TPA (MW 166 g mol^-1^) and EG (MW 62 g mol^-1^) per repeat unit. Carbon atoms are traced through each downstream pathway to verify elemental conservation. All yields represent theoretical upper bounds under idealized complete-conversion conditions. Realized yields will be substantially lower due to maintenance energy expenditure (which typically consumes 30–50% of the total carbon budget in metabolically engineered bacteria), competing catabolic routes, kinetic bottlenecks, and thermodynamic constraints. The carbon recovery values in Table 2 are therefore illustrative of the theoretical maximum flux through each pathway, not predictions of experimental outcome.

**Table 2:** Corrected theoretical stoichiometric yields of P2F fertilizer-grade compounds from PET degradation.

Product	Molar yield (mol per mol PET repeat unit)	Mass yield (g per g PET)	Carbon routed (% of PET C)	Agronomic function, stoichiometric basis, and notes	Reference
Citric acid	0.33	0.058	~10	Rhizosphere phosphate solubilization; soil acidulant. Routed via TCA citrate synthase; succinyl-CoA synthetase deletion (sucC) predicted to reduce competitive flux (pending FBA validation). Stoichiometric basis: Harwood and Parales; PET carbon basis from Yoshida et al.	Harwood and Parales (1996) and Yoshida et al. (2016)
Succinic acid	0.28	0.030	~7	Soil organic carbon source; acidulant. Fumarate reductase deletion predicted to favor succinate accumulation. Pathway basis: Harwood and Parales.	Harwood and Parales (1996)
Glutamate	0.18	0.024	~6	Slow-release nitrogen source (~9.5% N w/w). Produced via GDH overexpression or GS-GOGAT acting on glyoxylate-derived *α*-ketoglutarate. EG-derived carbon routing supported by Knott et al.	Harwood and Parales (1996) and Knott et al. (2020)
Aspartate	0.12	0.015	~4	Slow-release nitrogen source (~10.5% N w/w). Produced via AspC overexpression and tdcB deletion, directing oxaloacetate-derived nitrogen toward the aspartate family.	Harwood and Parales (1996)
Humic-like macromolecules (HLMs)	n/a (mass basis only)	0.08–0.12	~8–12	Enhances CEC and water retention; structural soil organic matter analogue. Formed via oxidative polymerization of phenylacetate and protocatechuate by CotA laccase and heterologous tyrosinase (MelB).	Santo et al. (2013)
CO₂ and H₂O (respiratory losses)	~5–7 mol CO₂ per PET repeat unit (theoretical)	~0.44	~61	Unavoidable metabolic by-products of central carbon metabolism. PET (C₁₀H₈O₄, 192 g/mol) yields ~84 g useful P2F outputs (35–45% C recovery) and ~108 g CO₂/H₂O equivalents. PET elemental composition from Yoshida et al.	Yoshida et al. (2016)

The PET repeat unit (C10H8O4; MW 192 g mol-1) is the stoichiometric basis (Yoshida et al., 2016). Carbon recovery percentages reflect theoretical maximum flux through each pathway. Total useful carbon recovery is estimated at approximately 35–45% of PET-derived carbon, with the remainder lost as CO2 and H2O through respiratory metabolism. All values are theoretical upper bounds; realized experimental yields will be substantially lower. Note on CO2 molar yield: the value of approximately 5–7 mol CO2 per repeat unit reflects variability across routing scenarios and carbon loss to maintenance energy; it is calculated from the difference between total PET carbon (10 carbons per repeat unit) and the carbon directed to useful P2F outputs (approximately 3–4.5 carbons), with the remainder lost as CO2. The earlier value of 2.8 CO2 per repeat unit appearing in a previous version of this manuscript was inconsistent with this balance and has been corrected. CEC, cation exchange capacity; HLM, humic-like macromolecule; TPA, terephthalic acid; EG, ethylene glycol; TCA, tricarboxylic acid cycle; GDH, glutamate dehydrogenase. Stoichiometric pathway basis: Harwood and Parales (1996), Knott et al. (2020), Santo et al. (2013), and Yoshida et al. (2016).

### 4.5 Modeling assumptions and limitations

**Figure 7:**
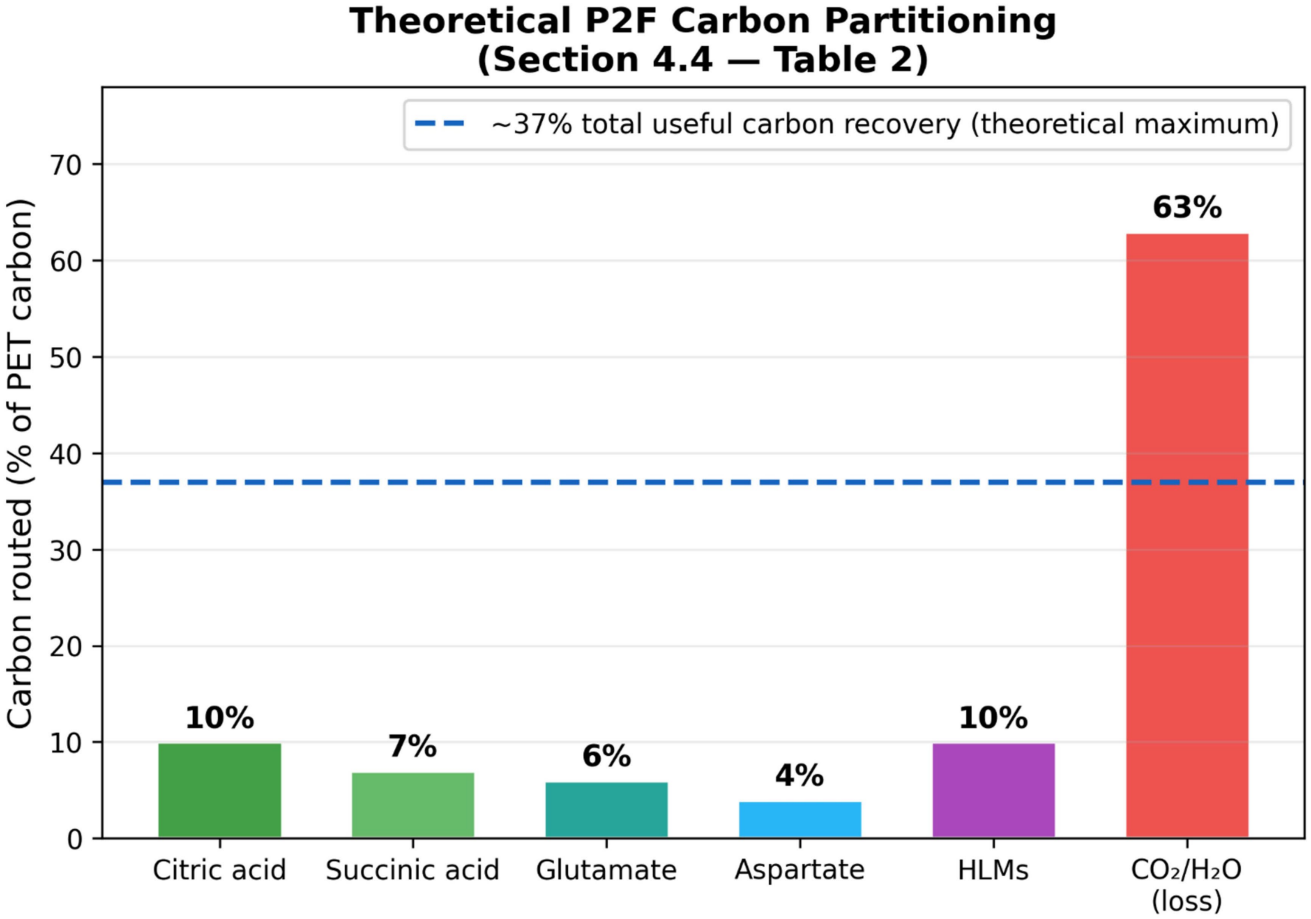
Theoretical carbon partitioning from PET degradation through the P2F metabolic funnel. Values derived directly from Table 2. Approximately 37% of PET-derived carbon is theoretically recoverable as useful P2F compounds (organic acids, amino acids, HLMs) under idealized conditions. The remaining ~63% is lost as CO₂ and H₂O through respiratory metabolism. All values are theoretical upper bounds; realized yields will be substantially lower as discussed in Section 11.1.

The theoretical simulations presented in Sections 4.1 to 4.4 are subject to the following explicit assumptions, which define the boundary conditions of the model predictions and must be addressed during experimental validation. For the surface degradation model (Section 4.1), it is assumed that enzyme adsorption follows ideal Langmuir behavior with a single binding site class, that the accessible surface area scales linearly with polymer mass (As = alpha × P), and that enzyme activity remains constant over the simulation period with no denaturation or inhibition by degradation products. In reality, post-consumer PET contains UV-degradation products, pigments, and plasticisers that may reduce enzyme activity significantly (Bell et al., 2022; Henawy et al., 2026). The surface exposure coefficient alpha is treated as a fixed value; experimentally it will vary with particle size distribution, crystallinity, and pre-treatment method. For the Haldane-Andrews growth model (Section 4.2), each organism is assumed to grow on a single primary substrate independently, with no cross-inhibition between consortium members and no spatial gradients in substrate concentration. Kinetic parameters (mu_max, Ks, Ki) are sourced from published monoculture studies (Wierckx et al., 2015; Poole et al., 2018; Cao et al., 2022) and may differ substantially in multi-species co-culture, where competition, cross-feeding, and quorum sensing interactions alter effective growth rates. The dilution rate D is held constant, which assumes stable bioreactor operation not yet demonstrated for this consortium. For the synergy model (Section 4.3), DS values are derived from Knott et al. (2020) under controlled laboratory conditions using purified enzymes and low-crystallinity PET films. DS values in the P2F system operating on post-consumer mixed plastic waste are expected to be lower due to enzyme fouling, product inhibition by contaminants, and non-ideal mixing. For the stoichiometric carbon balance (Section 4.4), all yields assume complete and exclusive channeling of carbon through the designated P2F funnel pathways with no competing reactions and no maintenance energy expenditure. As noted in Table 2, realized experimental yields will be substantially lower than the theoretical upper bounds presented here, with maintenance metabolism alone consuming an estimated 30 to 50% of available carbon (Cao et al., 2022). These assumptions are consistent with the TRL 1 to 2 status of the P2F framework. The primary purpose of the simulations is to generate falsifiable quantitative predictions, specifically the parameter ranges and outcome thresholds listed in Table 3, that will be tested systematically in Phases 1 through 3 of the experimental roadmap (Figure 7).

**Table 3:** Five-phase experimental validation roadmap for the P2F framework.

Phase	Focus	Key Experiments	Success Criteria	Timeline	Reference
**1**	Enzyme characterization in *P. putida*	Express FAST-PETase and MHETase in *P. putida* KT2440 via chromosomal integration; measure kcat and KM on crystalline and amorphous PET films by HPLC; screen AlmA variants on UV-pre-oxidized HDPE films by GC–MS; evaluate enzyme activity in presence of common plastic additives.	Measurable PET hydrolysis (>5 nmol TPA h^−1^ mg enzyme^−1^); detectable AlmA activity on pre-oxidized HDPE; >50% activity retention with additives.	Months 1–10	Bell et al. (2022), Henawy et al. (2026), and Mohanan et al. (2020)
**2**	Single-strain P2F funnel engineering	Chromosomal integration of P2F funnel modules F1–F3 into *P. putida*; measure organic acid and amino acid titres from TPA and EG feeding by HPLC; verify mass balance via carbon tracking.	Citrate >0.05 g g^−1^ TPA; glutamate detectable; no growth inhibition from funnel expression.	Months 6–22	Wierckx et al. (2015), Harwood and Parales (1996), and Knott et al. (2020)
**3**	Co-culture stability testing	Pairwise and four-species co-culture on UV-pre-oxidized plastic films; species ratio monitoring by qPCR; polymer mass loss by FTIR and gravimetry; consortium stability over 28 days.	Stable co-culture (>5% relative abundance for all members) for >21 days; >15% PET mass loss in 28 days; no styrene oxide above threshold.	Months 18–34	Cao et al. (2022) and Howard and McCarthy (2023)
**4**	Two-stage bioreactor prototype testing	Assemble modular two-stage bioreactor; feed mixed pre-treated plastics (PET: HDPE: PS = 50:30:20); measure P2F compound titres across three 14-day batches; 16S rRNA profiling for consortium stability.	Consistent P2F titres (<20% CV across batches); stable consortium composition; no GMM detected in effluent.	Months 30–46	Jadaun et al. (2022) and Henawy et al. (2026)
**5**	Agronomic efficacy trials	Apply spray-dried P2F product to *Arabidopsis thaliana* and *Zea mays* pot trials at two dose levels; measure NUE, biomass, chlorophyll content, and soil microbial diversity vs. urea and unfertilized controls.	NUE not significantly below urea (*p* > 0.05); no phytotoxicity at recommended dose; no significant reduction in soil microbial diversity.	Months 44–58	Hasan and Tarannum (2025)

Timeline estimates assume a well-resourced academic or industrial laboratory setting; actual timelines will vary. Each phase generates independently publishable data. Abbreviations: NUE, nitrogen use efficiency; qPCR, quantitative polymerase chain reaction; FTIR, Fourier-transform infrared spectroscopy; HPLC, high-performance liquid chromatography; GC–MS, gas chromatography–mass spectrometry; TFF, tangential flow filtration; rRNA, ribosomal RNA. Key references: Phase 1, Bell et al. (2022), Mohanan et al. (2020), and Henawy et al. (2026); Phase 2, Wierckx et al. (2015), Harwood and Parales (1996), and Knott et al. (2020); Phase 3, Cao et al. (2022) and Howard and McCarthy (2023); Phase 4, Jadaun et al. (2022) and Henawy et al. (2026); Phase 5, Hasan and Tarannum (2025).

## 5 Consortial dynamics and ecological stability

### 5.1 Metabolic complementarity and cross-feeding

The P2F consortium is designed so that metabolic outputs of each member serve as inputs for others, creating interdependencies that reduce competitive exclusion. *B. subtilis* and *A. niger* surface oxidation of PE and PP generates functionalized oligomers that serve as substrates for *P. putida* alkane monooxygenase activity. TPA and EG from PETase/MHETase activity in *P. putida* feed the beta-ketoadipate pathway and glyoxylate shunt, respectively. *A. vinelandii* assimilates small organic acids via TCA cycle enzymes, a process that requires aerobic respiration, while nitrogenase activity, if expressed, is conditional on the anoxic biofilm interior. It is acknowledged that this places the same organism in two physiologically distinct states simultaneously; resolving this tension will require direct experimental characterization of *A. vinelandii* oxygen consumption and growth behavior under the partial oxygen tensions expected within the consortium biofilm, and is identified as a priority question for Phase 3 co-culture experiments. The quantitative cross-feeding rates are not characterized and must be measured in pairwise and multi-species co-culture experiments (Phase 3) before the stability of this network can be assessed (Cao et al., 2022).

### 5.2 Niche partitioning within biofilm architecture

Chemical gradients of O2, pH, and substrate concentration within a biofilm impose spatial niche partitioning. *A. niger* is expected to dominate the outermost, oxygen-replete biofilm layer in direct contact with the polymer surface. *P. putida* and *B. subtilis* occupy the aerobic middle zone. *A. vinelandii* nitrogenase activity, if functionally expressed, requires the anoxic biofilm interior where dissolved O2 falls below the inhibitory threshold of approximately 0.1 micromolar. Synthetic adhesin-ligand pairs based on SpyCatcher/SpyTag surface display technology (Zakeri et al., 2012) may direct cell–cell attachment to reinforce this layered architecture, but this approach has not been validated in this consortium context and remains a design hypothesis. The coexistence of oxidative surface chemistry and anoxic microzones within the same biofilm is supported by well-characterized oxygen-diffusion dynamics in mixed microbial communities. In thick, polymer-attached biofilms, oxygen penetration is typically limited to the outer 50–150 μm, creating a stratified architecture in which aerobic organisms (*P. putida*, *B. subtilis*, *A. niger*) occupy the oxygenated periphery while respiratory oxygen consumption and extracellular polymeric substance (EPS) density generate micro-anoxic pockets in the interior. These microzones are transient and spatially heterogeneous, but they are sufficient to support conditional nitrogenase activity in *A. vinelandii*, which is known to exploit localized low-oxygen niches even within otherwise aerobic environments. Importantly, the oxidative reactions occur at the polymer–biofilm interface, whereas nitrogenase activity is restricted to deeper layers that do not compete for the same physical space. This spatial separation mitigates the apparent tension between oxidative and anoxic processes and is consistent with empirical observations of stratified biofilms in wastewater, soil aggregates, and mixed-species bioreactors. It should be acknowledged, however, that biofilm channel formation, which tends to develop in thicker or more mature biofilms, can disrupt exactly the kind of stratified oxygen gradient the P2F spatial model assumes by introducing convective oxygen transport into deeper layers. The P2F design therefore depends on maintaining a biofilm thickness regime in which diffusion-limited oxygen depletion dominates over channel-mediated mixing; this parameter will need to be characterized and controlled during Phase 3 co-culture experiments, and the assumed spatial architecture should be treated as a design hypothesis pending direct measurement of oxygen microprofiles within the consortium biofilm.

### 5.3 Population ratio control via synthetic quorum sensing

Synthetic quorum sensing (QS) circuits based on acyl-homoserine lactone (AHL) signaling in *P. putida* and AI-2 signaling in *B. subtilis* are proposed to maintain inter-species population ratios within a defined operating envelope. Circuit design principles follow established synthetic QS architectures reviewed by Cao et al. (2022). Lotka-Volterra stability analysis predicts a coexistence equilibrium for the nominal parameter range, but this prediction is conditional on kinetic parameters that must be determined experimentally; the stability condition is a design hypothesis requiring Phase 3 co-culture validation.

## 6 Theoretical reactor design

Three bioreactor configurations are considered for P2F consortium deployment. The modular two-stage system is identified as the preferred configuration based on process controllability, stage-specific optimization potential, and biocontainment manageability (Figure 3).

Biofilm Membrane Reactor (BMR): Shredded plastic particles (1–5 mm) are loaded into a packed-bed reactor where consortium biofilm colonizes the particle surface. Continuous tangential flow filtration (TFF) removes solubilized monomers for downstream processing. Advantages include high biomass retention and efficient solid–liquid mass transfer. Primary challenges are progressive packed-bed clogging and difficulty maintaining distinct spatial niche zones.

Sequential Fed-Batch System: Abiotic pre-treated plastic is batch-inoculated with the full consortium, with a surface oxidation and depolymerization phase (days 1–14) followed by a monomer conversion and biosynthesis phase (days 15–28). Simpler to operate but less amenable to stage-specific optimization.

Modular Two-Stage System (Preferred): Stage 1 is a solid-phase surface oxidation unit employing *A. niger* and *B. subtilis* biofilm on shredded, UV/ozone pre-treated plastic particles. Stage 2 is a liquid-phase continuous stirred tank reactor (CSTR) where *P. putida* and *A. vinelandii* convert solubilized monomers to P2F products. Physical separation allows independent optimization of each stage and simplifies biocontainment. O2-scavenging membrane panels in Stage 2 aim to maintain anoxic niches for conditional *A. vinelandii* nitrogenase activity.

## 7 Biosafety, containment, and regulatory considerations

### 7.1 Genetic containment strategy

Three independent containment layers are proposed. First, synthetic auxotrophy: all engineered strains require p-azido-L-phenylalanine, a non-natural amino acid absent from natural environments, for growth. This is achieved by engineering a UAG amber suppressor tRNA/aminoacyl-tRNA synthetase pair that incorporates p-azido-L-phenylalanine at an essential gene position; without the amino acid supplement, cell growth is impossible. Second, MazF-ATC toxin-antitoxin kill switch: a chromosomally integrated conditional lethality system causes cell death upon withdrawal of anhydrotetracycline (ATC). The combination of auxotrophy and kill switch provides two independent containment barriers whose simultaneous failure would require two statistically independent low-probability events. Third, genome recoding (long-term measure): reassignment of all TAG amber stop codons to TAA, combined with repurposing TAG to encode a synthetic amino acid, would prevent productive expression of horizontally acquired wild-type gene sequences, providing a third independent containment barrier.

### 7.2 Regulatory pathway

In the United States, GMM-based bioprocess agents require regulatory coordination among the EPA (TSCA Biotechnology Program), USDA (APHIS), and FDA prior to any agricultural soil application. Required documentation includes an Environmental Risk Assessment per OECD Guidelines 203–208, horizontal gene transfer probability assessments, contained greenhouse field trials, and a Life Cycle Assessment demonstrating net environmental benefit. In the European Union, Directive 2001/18/EC and Regulation (EC) No. 1829/2003 apply to deliberate release of genetically modified organisms (GMOs). The five-phase experimental roadmap (Section 10) is structured to generate the empirical data required for these regulatory submissions in sequence.

## 8 Comparative technology assessment

The P2F framework must be evaluated relative to established plastic management technologies to identify where it may offer advantages and where its current limitations are greatest (Table 4). The most significant potential advantage of the P2F approach is its dual-value proposition: simultaneous plastic waste processing and production of agronomically useful compounds. However, the TRL (1–2) of the P2F framework must be clearly acknowledged relative to commercially deployed technologies (Jadaun et al., 2022). This section provides a brief comparative technology assessment intended solely for conceptual framing at TRL 1–2. No experimentally validated techno-economic analysis is presented. Instead, the discussion highlights qualitative differences between biological plastic upcycling and conventional mechanical or chemical recycling routes. Biological conversion of PET, HDPE, PP, and PS into agronomically beneficial compounds offers potential advantages in carbon retention and product diversification, but current enzymatic and metabolic limitations place the technology at an early conceptual stage. All techno-economic considerations referenced in this manuscript are therefore illustrative, order-of-magnitude conceptual scenarios rather than quantitative predictions of process performance or cost. Future TEA will require experimentally measured kinetic parameters, validated yield data, and reactor-scale performance metrics. Until such data exist, any comparison to established recycling technologies should be interpreted as qualitative and exploratory.

**Table 4:** Comparative technology assessment of the P2F framework versus established plastic management technologies.

Parameter	Mechanical recycling	Chemical recycling	Composting	P2F framework	Reference
Plastic types handled	Clean, single-type only	Most thermoplastics	Certified biodegradable only	Mixed PET/HDPE/PS/PP streams (theoretical; TRL 1–2)	Geyer et al. (2017) and Mohanan et al. (2020)
Energy input	Moderate	High	Low	Low (metabolic); abiotic pre-treatment required and adds energy cost	Geyer et al. (2017)
Toxic by-products	Minor (additive leaching)	Yes (solvents, catalysts)	Minimal	Potential styrene oxide accumulation; mitigation via StyC overexpression + real-time HPLC monitoring	Wierckx et al. (2015)
Output value	Recycled polymer (quality decreases per cycle)	Monomers or fuel	Compost	Theoretical biofertilizer compounds (organic acids, amino acids, HLMs); market value unvalidated	Hasan and Tarannum (2025)
Mixed/contaminated stream tolerance	Poor	Moderate	Not applicable	Designed for mixed streams; contaminant-mediated enzyme inhibition is a recognized limitation	Mohanan et al. (2020) and Henawy et al. (2026)
Scale-up maturity	Commercial	Pilot to commercial	Commercial	Theoretical (TRL 1–2); proof-of-concept validation required at every stage	Cao et al. (2022)
Regulatory pathway	Established	Established	Established	Novel; GMM-specific approval required (EPA/USDA/FDA in USA; Directive 2001/18/EC; Regulation (EC) No. 1829/2003 in EU)	European Parliament and Council (2001) and European Parliament and Council (2003); U.S. Environmental Protection Agency (2026)
Net GHG vs. landfill	Moderate benefit (documented)	Moderate benefit (documented)	Moderate benefit (documented)	Uncertain; potentially favorable only if nitrogen fixation offsets Haber–Bosch production; high uncertainty at current TRL	Jadaun et al. (2022)

TRL, technology readiness level; GMM, genetically modified microorganism; GMO, genetically modified organism; GHG, greenhouse gas; HDPE, high-density polyethylene; PS, polystyrene; PP, polypropylene; PET, polyethylene terephthalate; HLM, humic-like macromolecule; HPLC, high-performance liquid chromatography. Data for established technologies from Jadaun et al. (2022) and Henawy et al. (2026). Regulatory information based on publicly available EPA, USDA, and EU regulatory frameworks (European Parliament and Council, 2001; Regulation (EC) no. 1829/2003, n.d.). Contaminant inhibition data from Mohanan et al. (2020). All P2F entries are theoretical at current TRL.

## 9 Risk analysis

Industrial deployment of engineered microbial consortia requires systematic risk characterization and pre-specified mitigation strategies. Table 5 presents a structured risk matrix for the P2F framework. The overall risk profile is comparable to other proposed GMM-based bioremediation systems (Cao et al., 2022). Horizontal gene transfer of the nif cluster represents the highest-priority biosafety concern given the ecological sensitivity of nitrogen fixation pathways; three independent mitigation mechanisms are proposed.

**Table 5:** Risk analysis matrix for the P2F framework. Likelihood and severity ratings are qualitative assessments based on analogous GMM bioprocess literature.

Risk Category	Specific Risk	Likelihood	Severity	Mitigation Strategy	Reference
Gene transfer	Horizontal transfer of *nif* cluster to indigenous soil bacteria	Low (contained reactor only)	High (if open release occurs)	Genome recoding; synthetic auxotrophy; terminators flanking *nif* locus; no environmental release until OECD 203–208 assessment	Jadaun et al. (2022)
Gene transfer	Transfer of antibiotic resistance markers	Low to moderate	Moderate	Antibiotic-free selection using synthetic auxotrophy; removal of all resistance cassettes post-construction	Cao et al. (2022)
Ecological	Displacement of indigenous soil microbial communities	Low (contained reactor)	High (if open release)	Strict reactor containment; ecological impact assessment before any open-environment application	Geyer et al. (2017)
Metabolic	Styrene oxide accumulation (reactive epoxide; potentially mutagenic)	Moderate	Moderate	Constitutive StyC overexpression; real-time HPLC monitoring; automated shutdown at styrene-oxide threshold	Wierckx et al. (2015)
Metabolic	Unintended nitrogen cycle perturbation from nitrogenase activity	Low to moderate	Moderate	Titratable *nif* expression; ammonium sensor-linked kill switch; nitrogen mass-balance tracking	Poole et al. (2018)
Process	Reactor breach releasing GMMs to environment	Low	High	Double-containment reactor; triple biocontainment (auxotrophy + MazF-ATC + genome recoding); daily viability monitoring	European Parliament and Council (2001)
Evolutionary	Loss of engineered constructs under sustained selection pressure	Moderate	Moderate	Chromosomal integration; growth-coupling of P2F funnel; periodic re-inoculation from validated cryostocks	Cao et al. (2022)
Engineering	Mass transfer limitations at pilot and commercial scale	Moderate	Moderate	CFD modeling of two-stage reactor; milestone-gated scale-up	Henawy et al. (2026)

CFD, computational fluid dynamics; HPLC, high-performance liquid chromatography; GMM, genetically modified microorganism; QS, quorum sensing. Mitigation strategy references: gene transfer biosafety (Jadaun et al., 2022); synthetic QS population control (Cao et al., 2022); nitrogenase oxygen regulation (Poole et al., 2018); evolutionary stability of engineered constructs (Cao et al., 2022).

## 10 Experimental validation roadmap

This framework is theoretical. Empirical validation requires a structured, milestone-gated pathway that identifies failure modes at the earliest and least costly experimental stage. The five-phase roadmap below translates theoretical objectives into testable hypotheses with quantitative success criteria (Table 3). Each phase is designed to yield independently publishable data regardless of whether the overall P2F system achieves its targets.

## 11 Discussion

### 11.1 Key limitations and uncertainties

The most fundamental limitation of the P2F framework is its exclusively theoretical nature. None of the enzymatic activities, metabolic yields, consortial dynamics, or reactor performance characteristics described here have been experimentally demonstrated in the proposed multi-organism, multi-substrate system. Five categories of limitation warrant particular emphasis.

Enzyme performance on real plastic waste: The kinetic models presented use parameter values from studies employing purified enzymes and laboratory-grade, low-crystallinity PET films. Post-consumer plastic waste streams contain UV-degradation products, plasticisers, pigments, and polymer blends that inhibit enzymatic activity through competitive and non-competitive mechanisms (Henawy et al., 2026). Phase 1 enzyme characterization using real waste fractions is therefore the most critical near-term experimental objective.

HDPE and PP degradation: As detailed in Section 2.2.2, intact semicrystalline polyolefins are not equivalent to the soluble alkane substrates on which AlkB and AlmA have been characterized. The absence of hydrolysable bonds in these polymers means that biological routes to depolymerization remain entirely dependent on prior abiotic pre-treatment steps, and the efficiency of those pre-treatment steps on realistic waste streams has not been quantified for the P2F context (Mohanan et al., 2020).

Nitrogen fixation module: The technical barriers to heterologous nitrogen fixation are well-documented and include oxygen sensitivity, ATP cost, metallocofactor assembly complexity, and tight regulatory requirements (Poole et al., 2018). This module is not treated as a near-term engineering deliverable; its absence would not compromise the core P2F concept, which generates soil-beneficial compounds regardless of whether biological nitrogen fixation is active.

Stoichiometric yield gap: Theoretical yields in Table 2 assume complete channeling of carbon through P2F funnel pathways with no competing reactions, no maintenance energy expenditure, and no thermodynamic losses. In practice, maintenance energy demands typically consume 30–50% of the total carbon budget in metabolically engineered bacteria, and competing catabolic routes further reduce product yields. Bridging this gap is the primary optimization objective for Phases 1 and 2 (Cao et al., 2022).

Evolutionary instability: Continuous culture imposes strong selection for mutants that shed metabolic burdens, including engineered P2F funnel enzymes. Chromosomal integration and growth-coupling strategies mitigate but cannot eliminate this risk (Cao et al., 2022). Mandatory regular phenotypic verification and cryostock re-inoculation protocols are therefore included in the operational design.

### 11.2 Positioning relative to existing approaches

The P2F framework occupies a conceptually distinct position from existing plastic management approaches, as summarized in Table 4. Comprehensive reviews confirm that no biological system has yet demonstrated efficient, scalable depolymerization of mixed plastic streams under ambient conditions (Henawy et al., 2026; Jadaun et al., 2022). The work of Cao et al. (2022) on consortium design for complex substrate degradation, and of Howard and McCarthy (2023) on biofilm-enhanced plastic-degrading enzyme activity, provide the most relevant experimental precedents for the consortium architecture proposed here. Multi-omics approaches cataloged by Henawy et al. (2026), including metagenomics, metatranscriptomics, metaproteomics, and metabolomics, represent informative tools for identifying additional plastic-degrading enzymes and characterizing consortium function *in situ*.

The key potential differentiator of the P2F framework relative to purely mineralizing approaches is the value proposition of converting plastic-derived carbon into soil-beneficial compounds. Organic acid-mediated phosphate solubilization in alkaline soils is a well-established agronomic mechanism (Hasan and Tarannum, 2025). Amino acid-based slow-release nitrogen sources have been associated with improved nitrogen use efficiency and reduced leaching compared to inorganic fertilizers, though direct comparisons with P2F-derived amino acid streams would need to be established empirically (Hasan and Tarannum, 2025). HLMs, if produced at sufficient yield and characterized for soil function, could provide cation exchange capacity and water retention benefits analogous to natural humic substances; however, direct empirical testing of P2F-derived HLMs on target soils and crops is essential before agronomic claims can be made.

### 11.3 Agricultural application context and limitations

**Figure 8:**
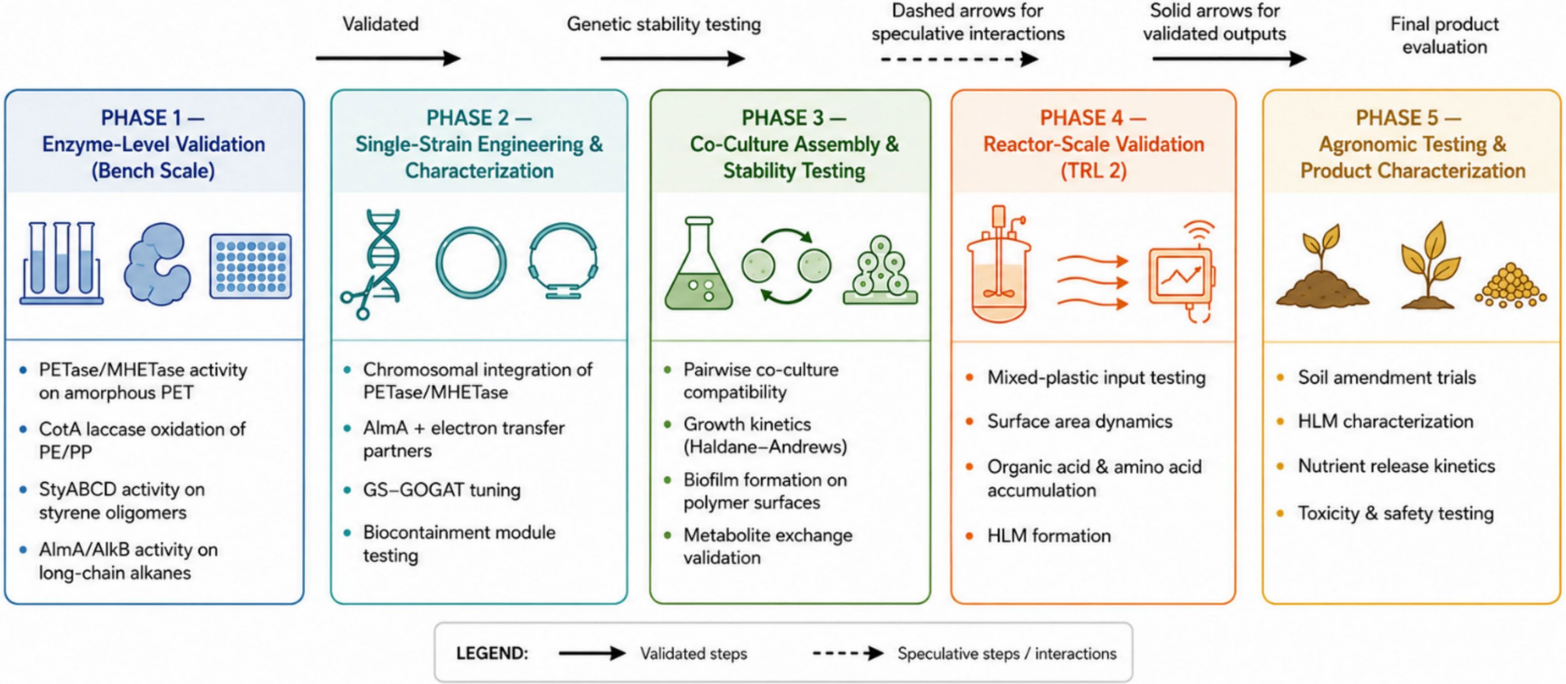
Five-phase experimental roadmap for P2F framework validation. Sequential validation pipeline from enzyme-level testing to agronomic evaluation. Solid arrows indicate experimentally validated steps; dashed arrows indicate speculative or unvalidated interactions. Icons and color coding represent distinct experimental phases. The roadmap integrates enzyme characterization (Bell et al., 2022; Santo et al., 2013), strain-level engineering (Zrimec et al., 2021), co-culture stability and growth kinetics (Harwood and Parales, 1996), reactor-scale testing of mixed-plastic degradation (Henawy et al., 2026), and agronomic assessment of organic acids, amino acids, and HLMs (Hasan and Tarannum, 2025).

The chemical heterogeneity of the P2F product stream, a mixture of organic acids, amino acids, and HLMs, may be agronomically advantageous, as natural humus is itself chemically complex and provides multiple concurrent soil functions. All product compounds are metabolizable or structurally beneficial organic molecules, and the framework is explicitly designed to avoid introducing plastic fragments or reactive toxic intermediates such as styrene oxide into agricultural soils. The potential negative impacts of microplastics on soil physicochemical properties and crop health underscore the importance of plastic-safe management strategies (Hasan and Tarannum, 2025). Nonetheless, the direct agronomic efficacy of P2F product streams must be assessed in controlled pot and field trials as specified in Phase 5 of the experimental roadmap before any agronomic performance claims can be substantiated. Regulatory approval for soil application of P2F-derived materials, which may be classified as GMM products depending on formulation, will require additional characterization and risk assessment steps beyond those described in Section 7 (Figure 8).

## 12 Techno-economic assessment–illustrative scenario only

Important note: The following techno-economic analysis (TEA) is presented strictly as an illustrative scenario to identify dominant cost drivers and to assess the qualitative conditions under which the P2F framework might approach economic viability if all technical objectives were successfully achieved. All numerical estimates carry high uncertainty. They are not financial projections, investment forecasts, or performance claims for the P2F framework at its current TRL 1–2. Values are derived from published analogous bioprocess cost data (Jadaun et al., 2022) and organic fertilizer market data (Hasan and Tarannum, 2025), and are offered solely to guide experimental development priorities. No value in this section or Table 6 should be cited as a validated P2F performance figure.

**Table 6:** Illustrative techno-economic scenario for the P2F framework at a hypothetical 100 tonne plastic per year pilot scale.

Parameter	Illustrative estimate	Basis and key assumptions	Uncertainty level	Reference
Capital cost (100 t plastic yr^−1^ pilot)	~$2–5 million USD	Analogous small-scale bioprocess pilot facilities; includes two-stage reactor, TFF, spray dryer; no site-specific engineering data at TRL 1–2	High (±50%)	Jadaun et al. (2022)
Operating cost (energy and utilities)	~$80–150 per tonne	Aeration, mixing, temperature control; assumed lower than pyrolysis; no empirical P2F data	Moderate (±30%)	Geyer et al. (2017)
Operating cost (microbial and consumables)	~$20–40 per tonne	Growth media, non-natural amino acid, cryostock maintenance, monitoring labour	Moderate	Jadaun et al. (2022)
Biofertilizer product market value	~$200–400 per tonne	Based on specialty organic fertilizer markets; varies by region, purity, regulation	High (market-dependent)	Hasan and Tarannum (2025)
P2F fertilizer yield (conservative)	~250–350 kg per tonne PET	Based on corrected stoichiometry; assumes 40–50% realization of theoretical upper bound	High	Yoshida et al. (2016)
Illustrative break-even vs. landfill	Potentially favorable where tip fees >$80 per tonne	Landfill fees ~$50–150; rough arithmetic based on PET product value and avoided tip fees	High uncertainty	Geyer et al. (2017)
Illustrative break-even vs. mechanical recycling	Not competitive at current TRL	Mechanical recycling cost ~$50–100 per tonne; P2F requires major cost reductions	Moderate	Mohanan et al. (2020)
Key cost reduction levers	Enzyme titre; consortium productivity; co-location; purification	Not individually quantified; sensitivity analysis needed after Phase 1–2	High	Cao et al. (2022)

All values are indicative only and carry high uncertainty. No value should be cited as a performance claim for the P2F framework. TFF, tangential flow filtration; TRL, technology readiness level; GMM, genetically modified microorganism. Analogous bioprocess cost basis from Jadaun et al. (2022). Organic fertilizer market comparator data from Hasan and Tarannum (2025). Stoichiometric yield basis from Table 2 (this work).

Four key assumptions underpin this illustrative scenario: (i) all five experimental roadmap phases are completed successfully; (ii) enzyme titres and consortium productivities approach those of mature industrial bioprocesses, typically requiring multiple rounds of strain improvement; (iii) the P2F product is successfully positioned in the specialty organic fertilizer market at the estimated price range; and (iv) the GMM regulatory approval pathway is successfully navigated, adding substantial time and cost to development. Each assumption carries high uncertainty, and the overall TEA outcome is sensitive to all four.

Under this illustrative scenario, break-even relative to landfill may be approached in specific market niches with high tip fees (above approximately $80 per tonne) and accessible organic fertilizer markets. A rough arithmetic illustration: at a conservative P2F yield of 300 kg product per tonne PET and a product value of $300 per tonne, gross product revenue is approximately $90 per tonne PET. Combined with an avoided landfill tip fee of $80 per tonne, total gross income is approximately $170 per tonne, compared with estimated operating costs of $100–190 per tonne. Break-even is therefore plausible but not assured, and depends critically on product value, local tip fee, and enzyme/consortium productivity. The P2F framework is not cost-competitive with established mechanical recycling for clean plastic streams at current TRL. A formal sensitivity analysis should follow once Phase 1–2 experimental data constrain the primary parameters.

## 13 Future directions

### 13.1 AI-assisted enzyme engineering

Deep learning protein structure prediction (AlphaFold2, ESMFold) and generative protein design algorithms (ProteinMPNN, RFdiffusion) offer opportunities for rational enzyme engineering beyond the reach of conventional directed evolution (Zrimec et al., 2021; Henawy et al., 2026). For the P2F framework, AI-assisted design is particularly relevant for generating PETase variants with improved activity on crystalline, UV-degraded, or additive-contaminated PET surfaces, and for generating AlkB/AlmA variants with extended chain-length tolerance. Experimental validation on polymer substrates remains mandatory and cannot be replaced by computational screening alone (Henawy et al., 2026).

### 13.2 Directed evolution of consortium members and cross-feeding networks

Continuous directed evolution platforms, including phage-assisted continuous evolution (PACE) and OrthoRep orthogonal replication systems, enable *in vivo* mutagenesis and selection of metabolic pathways over many generations (Cao et al., 2022). Applied to P2F funnel modules under selection pressure for product titre, these platforms could identify beneficial genetic combinations not accessible to rational design. Evolution of inter-species cross-feeding under consortium-level selection is a particularly promising frontier, as emergent cooperative metabolic phenotypes have been observed in experimentally evolved co-cultures (Cao et al., 2022).

### 13.3 Genome-scale metabolic modeling and digital twin development

Simplified pathway balances underlie the stoichiometric estimates in Table 2. Genome-scale metabolic models (GEMs) are available for *P. putida* (iJN1411), *B. subtilis* (iYO844), and *A. vinelandii*, and could in principle be integrated into a consortium-level multi-organism flux balance analysis (mo-FBA) framework. Coupling such a model to spatial biofilm simulations such as iDynoMiCS and real-time reactor sensor data would yield a predictive digital twin for P2F bioreactor operation, enabling process optimization and model-informed regulatory submissions. GEM-based predictions must be validated experimentally before they can guide engineering decisions.

### 13.4 Multi-omics integration for consortium characterization

The multi-omics approaches catalogued by Henawy et al. (2026), including comparative genomics, metatranscriptomics, metaproteomics, and metabolomics, are informative tools for characterizing plastic-degrading microbial communities *in situ* without dependence on pure culture isolation. Applied to the P2F consortium during Phases 3 and 4 of the experimental roadmap, these approaches would enable identification of enzymatic bottlenecks, unexpected metabolic interactions between consortium members, and evolutionary trajectories that could compromise stability. The resulting datasets would provide the empirical basis for iterative refinement of the mathematical framework.

### 13.5 Substrate range extension and climate context

The P2F framework is not inherently restricted to the four plastic types addressed here. Extension to agricultural polyethylene mulch films, expanded PS packaging, and nominally biodegradable plastics, including polylactic acid (PLA) and polyhydroxyalkanoates (PHAs) whose degradation rates in agricultural soils are often overestimated, represents a natural progression. The concept of channeling plastic-derived carbon into stable soil organic matter forms rather than emitting it as CO2 aligns with biological carbon sequestration strategies (Hasan and Tarannum, 2025). However, carbon sequestration claims for the P2F framework should not be made until the persistence and soil stability of P2F-derived HLMs and organic acids have been experimentally determined under relevant field conditions.

## 14 Conclusion

This paper presents a rigorously qualified theoretical framework for an engineered four-member microbial consortium designed to convert mixed plastic waste into soil-beneficial fertilizer-grade compounds via the P2F concept. The principal contributions are: a biologically consistent consortium architecture with explicitly defined enzymatic roles, substrate constraints, and organism rationale; a corrected stoichiometric mass balance grounded in PET repeat unit elemental composition and published pathway stoichiometry; a mathematically coherent kinetic framework that correctly names and formulates the Haldane-Andrews growth equation and corrects a dimensional inconsistency in the surface degradation kinetic model; an explicit correction of the glutamate versus aspartate pathway logic in the F2 funnel module; a corrected CO2 molar yield estimate consistent with the stated carbon recovery values; realistic and conservative framing of HDPE, PP, and PS degradation claims; an honest treatment of the nitrogen fixation module as a speculative long-term goal; a clearly labelled illustrative TEA scenario with an arithmetic illustration of break-even logic; and a five-phase experimental validation roadmap with quantitative success criteria and explicit literature references.

The central conceptual contribution is the proposal to treat plastic polymer carbon as a feedstock for soil organic matter restoration rather than waste to be mineralised. The PET depolymerization and aromatic catabolism modules represent the most technically mature elements and constitute the near-term experimental priority. The framework is offered as an invitation to the synthetic biology, environmental microbiology, bioprocess engineering, and agronomy communities to engage with a multi-benefit approach to plastic waste valorization through stepwise, milestone-gated empirical validation.

## Data Availability

The original contributions presented in the study are included in the article/supplementary material, further inquiries can be directed to the corresponding author.
